# The crosstalk: exosomes and lipid metabolism

**DOI:** 10.1186/s12964-020-00581-2

**Published:** 2020-08-03

**Authors:** Wei Wang, Neng Zhu, Tao Yan, Ya-Ning Shi, Jing Chen, Chan-Juan Zhang, Xue-Jiao Xie, Duan-Fang Liao, Li Qin

**Affiliations:** 1 School of Pharmacy, Hanpu Science and Education District, Hunan University of Chinese Medicine, 300 Xueshi Road, Changsha, 410208 Hunan China; 2 Division of Stem Cell Regulation and Application, Hunan University of Chinese Medicine, Changsha, Hunan China; 3 The First Affiliated Hospital, Hunan University of Chinese Medicine, Changsha, Hunan China; 4 Department of Neurosurgery in Changsha, 921 hospital, joint service support force of People’s Liberation Army, Changsha, China; 5 College of Chinese Medicine, Hunan University of Chinese Medicine, Changsha, Hunan China

**Keywords:** Exosome, Lipid metabolism, Atherosclerosis, Cancer

## Abstract

Exosomes have been considered as novel and potent vehicles of intercellular communication, instead of “cell dust”. Exosomes are consistent with anucleate cells, and organelles with lipid bilayer consisting of the proteins and abundant lipid, enhancing their “rigidity” and “flexibility”. Neighboring cells or distant cells are capable of exchanging genetic or metabolic information via exosomes binding to recipient cell and releasing bioactive molecules, such as lipids, proteins, and nucleic acids. Of note, exosomes exert the remarkable effects on lipid metabolism, including the synthesis, transportation and degradation of the lipid. The disorder of lipid metabolism mediated by exosomes leads to the occurrence and progression of diseases, such as atherosclerosis, cancer, non-alcoholic fatty liver disease (NAFLD), obesity and Alzheimer’s diseases and so on. More importantly, lipid metabolism can also affect the production and secretion of exosomes, as well as interactions with the recipient cells. Therefore, exosomes may be applied as effective targets for diagnosis and treatment of diseases.

Video abstract

Video abstract

## Background

Exosomes display cup-like shape of 30 ~ 100 nm in diameter, and are secreted by multi-type cells, such as nerve cells [1], natural killer cells [2, 3], cancer cells [4, 5] and adipocytes [6]. Cell-secreted exosomes are transmitted into blood, amniotic fluid, urine, breast milk, cerebrospinal fluid, saliva, lymph and bile [7], and then interact with the receptor-ligand, internalize or fuse with the target cell membrane to send their own content into their cytosol, altering the physiological or pathological state of the recipient cell. Exosomes perform the parent cell-like behavior, because their structure or contents, consisting of lipids, proteins and nucleic acids, are derived from parent cells. For example, mastocyte-derived exosomes are rich in more sphingomyelin and phosphatidylethanolamine on the membrane [8]. Similar to the cell membrane, the lipid bilayer protects exosome contents from various stimuli in the circulating fluid. Therefore, some contents in exosomes are usually transported remotely in circulating body fluids, which exerts effects in physiological and pathological processes.

Notably, bioactive molecules in exosomes play significant roles on lipid transporters (e.g. ATP-binding cassette transporter A1 (ABCA1), ATP-binding cassette transporter G1 (ABCG1), CD36, low density lipoprotein receptor (LDLR) etc.), nuclear transcription factors (e.g. peroxisome proliferators-activated receptors (PPARs)), fatty acid synthetase (FASN) etc.) [9–13], further affect inflammatory response, immunology processes as well as cell apoptosis [14, 15], and ultimately leading to diseases related to lipid metabolism disorder, such as atherosclerosis, cancer, NAFLD, obesity, Alzheimer’s disease .

Conversely, increasing evidence indicated that lipid metabolism also affects biological processes of exosomes, including biosynthesis and interactions with recipient cells, which probably because lipids are the major components of bio-film systems and affect their fluidity. It has been confirmed that ABCA1-mediated cholesterol efflux can promote the release of exosomes, while SR-B1-mediated cholesterol efflux can inhibit the absorption of exosomes by recipient cells [16]. However, the relationship between exosomes and lipid metabolism is still unclear.

## The structure, composition, biofunctions and pathology of exosomes

### The structure characteristics

**Fig. 1:**
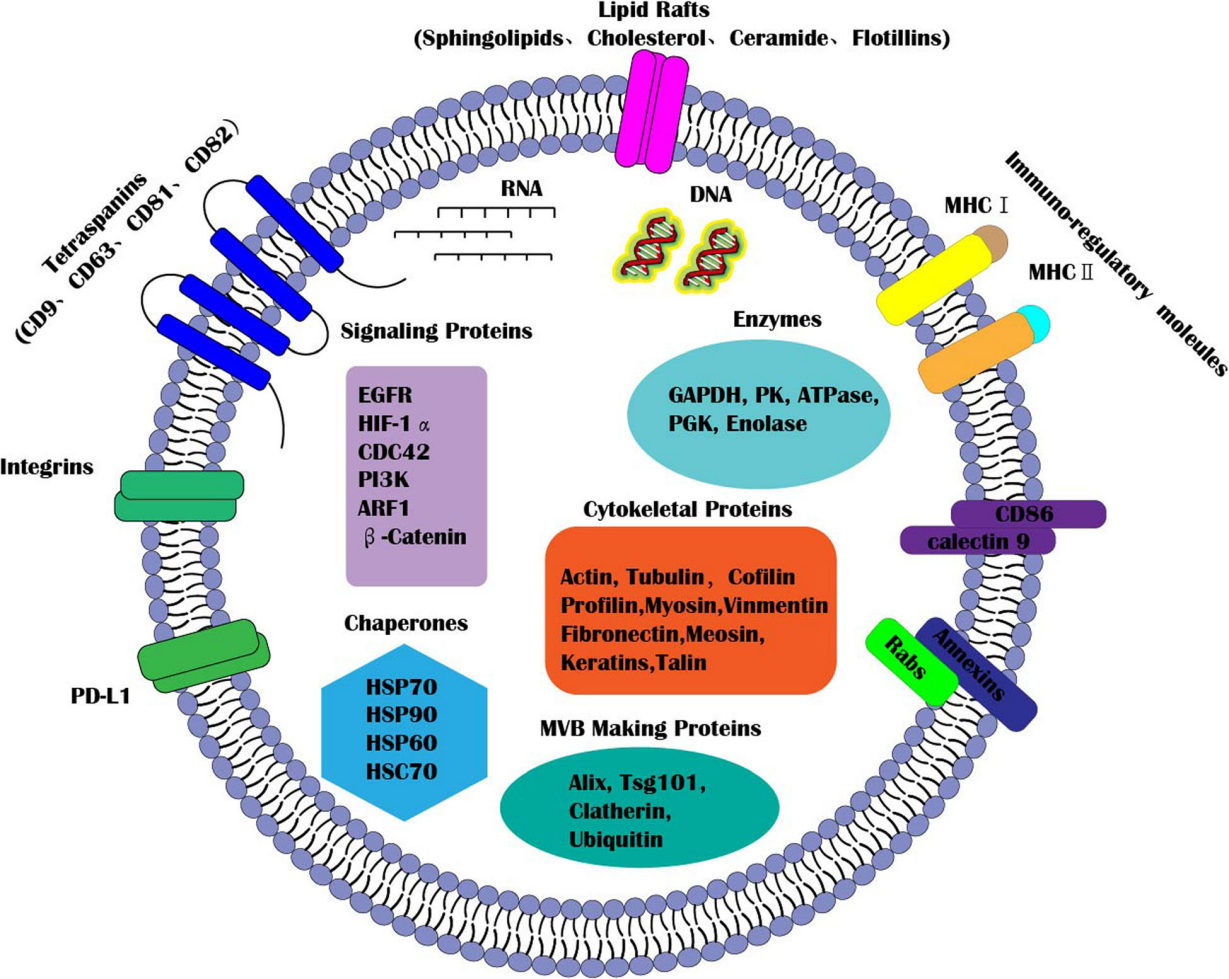
The basic structure and composition of exosomes. Exosomes have a typical lipid bilayer that protects and transfers exosomal bioactive molecules including proteins, lipids and nucleic acids. Exosomes from cells of different types have common proteins that can be used as cell surface markers such as annexins, flotillins, clathrin, Alix, TSG101, integrin and tetraspanins (CD63, CD9, CD81 and CD82). However, exosomes from specific sources have their own special markers, such as MHC-I/II on the surface of exosomes derived from dendritic cells, PD-L1 on the surface of cancer cell-derived exosomes, and adiponectin on the surface of adipocyte-derived exosomes. In addition, specific exosomes secreted by different cells also have their own specific compositions. In general, the proteins in exosomes can be divided into five categories, including signaling proteins (EGFR, HIF-1α, CDC42, PI3K, ARF1, β-Catenin), enzymes (GAPDH, PK, ATPase, PGK, Enolase), cytoskeletal proteins (Actin, Tubulin, Cofilin, profiling, Myosin, Vinmentin, Fibronectin, Meosin, Keratins, Talin), chaperones (HSP70, HSP90, HSP60, HSC70) and MVB making proteins(Alix, Tsg101, Clatherin, ubiquitin). Moreover, exosomes also carry many nucleic acids, including RNA and DNA

Generally, exosomes are consistent with anucleate cells, and organelles with lipid bilayer that helps to enhance their rigidity and flexibility (Fig. 1) [17]. On the one hand, the tail of the fatty acid oscillates the entire phospholipid molecules laterally, showing “flexibility”. On the other hand, cholesterol helps to maintain the structural stability and the arrangement of phospholipid bilayers, showing “rigidity”. In addition, exosome-surface proteins may also play a vital role in rigidity.

### The composition characteristics

Endosomes encapsulate annexins [18], flotillins [19], Rab molecules [20] and clathrin [21], etc., which play an important roles during the membrane fusion and transportation of exosomes. Tetraspanins (including CD9, CD63, CD81 and CD82) are mainly involved in the exosome formation [22, 23]. Alix [24], TSG101 [25], heat shock protein 70 (HSP70), and HSP90 [26] participate in the biosynthesis of multi-vesicular bodies (MVBs). It seems that the characteristic structural proteins of exosome include CD63, CD81, CD9, CD82, Alix, TSG101 and flotillins, which are regarded as markers of exosomes (Fig. 1).

Exosomes from different cells have their own signatures. For instance, platelet-derived exosomes contain bioactive prostaglandins and leukotrienes, while adiponectin is specific for adipocyte-derived exosomes [27], dendritic cell-derived exosomes are present in MHC-I/II [28], and PD-L1 is exist in cancer cell-derived exosomes [29]. Some receptor proteins exist on the surface of exosomes, such as platelet-derived growth factor receptors (PDGFR) [30], endothelial growth factor receptors (EGFR) [31], etc. Exosomes secreted by different types of cells may contain characteristic molecules with typical physiological and pathological functions. More importantly, some signaling composition are the basis for exosomes to exert physiological and pathological effects, such as molecules(e.g. HIF-1α [32], PI3K, ARF1 and β-Catenin), enzymes(e.g. GAPDH, PK, ATPase, PGK, etc.) and backbone proteins (Alix, TSG101, Clatherin, etc.). Some genetic materials in exosomes, such as various amounts of DNA (e.g. double-stranded DNA, single-stranded DNA, mitochondrial DNA) and RNA (e.g. messenger RNA, microRNAs, small nuclear RNA, non-coding RNA, small cytoplasmic RNA), can alter the expression of genetic information in target cells (Fig. 1) [33, 34]. Although exosomal microRNAs and lncRNAs have been extensively studied, exosomal DNAs receive little attention.

Nowadays, the lipid compositions in different cell-derived exosomes have been gradually explored [35]. For example, exosomes produced in colorectal cancer cell line LIM1215 have higher levels of sphingolipids, cholesterol, glycerides, and glycerophospholipids, especially plasmalogen and glycerol phosphate than parental cells [36]. In addition, exosomes derived from prostate cancer cells are rich in high glycosphingolipids, sphingomyelin, cholesterol, and phosphatidylserine [37–39]. Therefore, the altered lipids in exosomes might be helpful in the diagnosis and treatment of diseases.

### Biological and pathological functions of exosomes

Since exosome was formally named by Johnstone in 1987, it plays a vital role in physiological and pathological processes in both prokaryotes and eukaryotes, and no longer recognized as “cell dust” [40]. Exosome exerts its versatile effects on fundamental biological processes in pleiotropic manners, such as directly activating cell surface receptors via protein and bioactive lipid ligands, fusing their membrane contents into recipient cell plasma membrane and delivering effectors including transcription, oncogenes, small and large non-coding regulatory RNAs into recipient cells [41]. As a result, exosomes participate in stem cell maintenance [42], tissue repair [43], immune surveillance [44] and blood coagulation [45]. Regarding to their fundamental role in regulating biological processes, it is not surprising in some cases that exosomes are involved in the pathogenesis of diseases, such as inflammation [46], immunosuppression [47], cell apoptosis, lipid accumulation etc. Exosomes thus are regarded as signal packets: multifunctional signaling complexes for regulation of biological and pathological functions.

## The effects of exosomes on lipid metabolism

Recent findings have verified that exosomes act as biological vehicle and directly transfer lipids such as cholesterol, fatty acids and eicosanoids. Some enzymes (proteins) are also encapsulated in exosomes and are involved in lipid metabolism. In particular, miRNAs in exosomes have received more attention. Exosomes protects themselves from enzyme ribonuclease through the miRNAs they carry. Therefore, lipids and its modifying proteins, enzymes and miRNA can crosstalk with exosomes.

### Exosomes and lipid synthesis

As we known, the biosynthesis of fatty acid and cholesterol is a huge energy-consuming process that requires a large amount of acetyl-CoA, ATP, and oxygen. Disorder of fatty acid synthesis in liver leads to hepatocyte diseases, such as hepatocyte injury and inflammation [48]. PPAR-γ, a member of the nuclear super-receptor family, can strictly regulate lipid intake, storage and metabolism in the liver through the transcription of metabolic related genes. More importantly, there is no single nuclear transcription factor that can induce the adipose-cell differentiation in the absence of PPAR-γ [49, 50]. Some circulating miRNAs in exosomes, such as miR-155 and miR-27, are able to inhibit the PPAR-γ expression by binding to 3′ untranslated regions (3’URT) of target genes [51–53]. Among them, miR-122 belongs to abundant liver-specific miRNAs. It has been revealed that the 3′ UTR of up-regulated genes, such as fatty acid synthase (FASN) and acetyl-CoA carboxylase (ACC), are highly enriched in miR-122 recognition motifs, thereby increasing the biosynthesis of fatty-acid and cholesterol in liver [11, 54].

It is worth noting that the exosomes released by hypoxic adipocytes are enriched in enzymes related to de novo lipogenesis, such as ACC, glucose-6-phosphate dehydrogenase (G6PD), and FASN. Hypoxia-derived exosomes promoted lipid accumulation in recipient 3 T3-L1 cells compared to those lipids produced under normoxic conditions [12] (Fig. 3). Meanwhile, glycosylphosphatidylinositol (GPI)-anchored proteins released from adipocyte-derived exosomes into small adipocytes, increasing esterification, reducing triacylglycerol fatty acids and promoting the formation of lipid droplets [55].

### Exosomes and lipid transportation

Recent studies showed that exosomes are capable of directly transporting lipids from parent cells to recipient cells, such as, cholesterol, fatty acids, eicosanoids et al., which may cause the inflammation, immune or metabolism changes [15, 56, 57]. Wang et al. also demonstrated that during the development of Alzheimer’s disease, astrocytes-derived exosomes carry ceramide, and the accumulation of ceramide leads to neuronal apoptosis [1]. However, a growing number of reports provide striking and convincing evidence that exosomes can regulate the expression of classical lipid transporters, such as ABCA1, ABCG1, LDLR, CD36 (Fig. 3).

Reverse cholesterol transport (RCT) is the only mechanism that clears cholesterol in the body and has positive significance for maintaining cholesterol homeostasis. ABCA1, ABCG1 are primarily involved in the RCT. Specific knockout of ABCA1 or ABCG1 in macrophages and vascular smooth muscle cells (VSMCs) respectively promotes foam cell formation in LDLR ^−/−^ mice due to deficient cholesterol efflux. Some circulating miRNAs in exosomes, such as miR-30e and miR-92a, display inhibitory effects on ABCA1 and ABCG1, causing the intracellular accumulation of cholesterol [58]. Exosomes with HIV-1 protein Nef (exNef) can be rapidly uptake by macrophages, and subsequently release exNef into cells, resulting in down-regulation of ABCA1, reduction of cholesterol efflux and a sharp elevation of in the abundance of lipid rafts through reducing the activation of small GTPase Cdc42 and decreasing actin polymerization exosomes with exNef can be rapidly uptake by macrophages, rapidly, and then exNef is released into the cells, resulting in down-regulation of ABCA1 and inactivation of small GTPase Cdc42 as well as reduction of actin polymerization [59].

To balance the level of plasma cholesterol, cholesterol in VSMCs, monocytes, macrophages, or hepatocytes is usually absorbed through LDLR [60]. LDLR^−/−^ mice with administration of high-fat diet (HFD) showed cholesterol accumulation in macrophages on the blood vessel wall [61]. However, by treating with exosomes isolated from supernatants of macrophages exposed to lipopolysaccharides, hepatocytes are in the state of inflammation through the LDLR pathway. Exosomes from HFD-visceral adipose tissue can facilitate the formation of macrophage-derived foam cells by down-regulating ABCG1 expression [10].

Several studies also have shown that exosomes inhibit cholesterol absorption in macrophages not by competitive CD36 ligands, but by drastical reduction of total macrophage CD36. Exosomes derived from platelet can inhibit athero-thrombotic processes by reducing CD36-dependent lipid loading of macrophages and suppressing platelet thrombosis. Srikanthan et al. also confirmed that exosomes can increase protein ubiquitination and enhance proteasome degradation of CD36 [62]. In addition, Ramakrishnan et al. found the inhibitory effect of exosomes on endothelial cell proangiogenic responses by activating a CD36-dependent signal pathway, and deletion of CD36 impairs exosome-induced inhibition of microvascular endothelial cell migration [63].

### Exosomes and lipid degradation

It is known that white adipose tissue is mainly responsible for storing energy. The degradation of white adipose tissue provides sufficient energy for cell growth and proliferation, especially in cancers and cancer-associated cachexia. Lewis lung carcinoma (*LLC*)-derived exosomes have higher levels of phospho-hormone sensitive lipase (P-HSL, a marker of activated lipolysis). 3 T3-L1 adipocytes exposed to LLC-exosomes exhibit higher levels of glycerol release and lower levels of lipid droplets [64]. Sagar et al. showed that pancreatic cancer (PC)-derived exosomes contain adrenomedullin, a 52 amino acid peptide that ubiquitously expressed in adipocytes and can induce lipolysis [65]. Lung cancer exosomes can be internalized by human adipose tissue-derived mesenchymal stem cells (hAD-MSCs) and significantly inhibited the adipogenesis of hAD-MSC. Specifically, the TGFβ signaling pathway is involved in the inhibition of hAD-MSC adipogenesis by lung cancer exosomes [66]. Meanwhile, Khalyfa et al. found that exosomes derived from obstructive hypoventilation syndrome induced a significant increase in adipocyte lipolysis [67].

PPAR-α, another member of the nuclear super-receptor family, mainly involves in the degradation of fatty acid. Some circulating miRNAs in plasma, such as miR-122, miR-192, miR-27a-3p and miR-27b-3p, also inhibit the expression of PPAR-α by binding to 3’URT, further inhibit lipid degradation in white adipose tissue of obesity-related patients [9] (Fig. 3).

## The effects of lipid metabolism on biosynthesis, secretion and biofunction of exosomes

### The effects of lipid metabolism on exosome biosynthesis and secretion

**Fig. 2:**
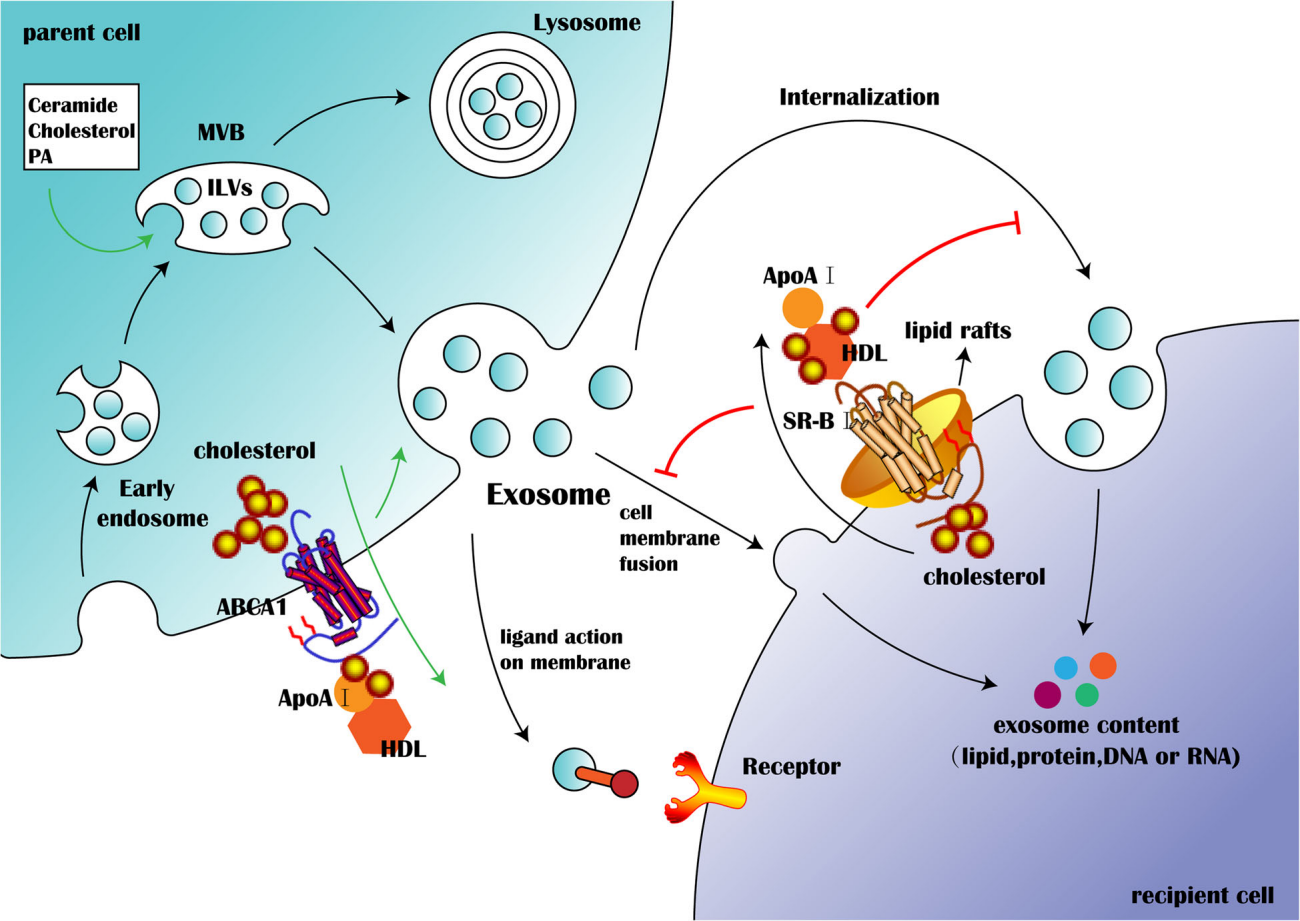
Lipid metabolism is involved in the biosynthesis and releases of exosomes, as well as its interaction with recipient cells. The transformation process from early endosomes to MVBs is induced by ceramide, cholesterol and PA. Then MVBs are either fused with lysosomes or with plasma membrane. And the fusion of MVBs with plasma membrane is promoted by ABCA1-mediated cholesterol efflux from the parent cells. The released exosomes deliver their contents to recipient cells through internalization, fusion with cell membrane or interaction with ligand on membrane. However, SR-B1-mediated cholesterol efflux inhibits the absorption of exosomes by recipient cells can be inhibited by SR-B1-mediated cholesterol efflux

The biological processes of exosomes from biosynthesis to release are complicated. The plasma membrane is inwardly recessed to form an early endosome [68], and the endosome then recesses exponentially and forms a MVB containing a large vast vesicles called intraluminal vesicles (ILVs). The fate of MVBs can be either fusion with lysosomes or fusion with plasma membrane. Given MVB is fused with the plasma membrane, the internal ILVs are released from the cells, so the extracellular ILVs are called exosomes (Fig. 2). ILVs are formed in the acidic environment (pH 5.5) of MVB and then released into the extracellular neutral pH environment to become “exosomes” [8]. Thereby, the differences between ILVs and exosome are more than just a name.

In mammalian, The main formation mechanism of exosomes is the endosomal sorting complex required for transport (ESCRT) [69], which relies on the ESCRT-associated proteins, such as TSG101, ALIX, VPS4 and tetraspanin etc. However, more lipids can enhance exosome secretion in the absence of ESCRT-associated proteins. The reduction of ceramide [22], cholesterol, and phosphatidylcholine (PA) in MVBs could decrease the secretion of exosomes. In addition, phospholipase D2 (PLD2) [70], diacylglycerol kinase (DGK) [71] and neutral sphingomyelinase [72] are also participated in this process. PLD2 and DGK are involved in the activation of PA, and neutral sphingomyelinase is involved in the activation of ceramide. The endosomes contain a group of monomolecular GTPases that activate PLD2, and PLD2 in both endosomes and exosomes activates PA that promotes the formation of ILVs. Hafiane and Genest et al. demonstrated the formation of exosomes during the process of ABCA1-dependent cholesterol efflux in different cell types [73]. In primary human monocyte-derived macrophages, apoA-I markedly increases release of exosomes. In contrast, inhibition of ABCA1 or decreasing plasma cholesterol dramatically reduced exosomes release (Fig. 2). However, Tafelmeier et al. showed that CD36 and SR-B1 mediate more efficient phospho- and sphingolipid remodeling in the absence of ABCA1 on platelet [16]. At present, there is little evidence that lipids play a key role in the formation and release of exosomes, and it has not been fully understood and requires further explored.

### The effects of lipid metabolism on exosomes biological functions

Exosomes transmit signals to recipient cells by receptor-ligand interaction, internalization or fusion with the membrane of recipient cells [74]. Some new ligands, such as integrin, PD-L1, and adiponectin, exposed on the surface of exosomes should rely on high lipids to exhibit its biological function. In mammalian, clathrin-dependent internalization is major internalization of exosomes. However, exosome internalization also occurs in the absence of clathrin, and the clathrin-independent internalization relies on cholesterol and tyrosine kinase activity [75]. Additionally, exosome internalization was inhibited by siRNA-mediated knockdown of caveolin-1, flotillin-1, RhoA, Rac1 and PAK1. Recently, Hazan-Halevy et al. also found that exosome internalization was mediated by a cholesterol-dependent pathway [76]. Probably more lipids enhance the fluidity of the membrane. In addition, it has been reported that internalization of receptor-ligand complexes is required for scavenger receptor CD36 [77], such as exosome internalization by monocytes [78] and hepatic macrophages [79]. Recently, Plebanek et al. demonstrated that the exosome internalization depends on lipid rafts, a cholesterol-rich membrane micro-domains, and also can be blocked by non-specific depletion of plasma membrane cholesterol. Further exploration showed that scavenger receptor type B-1 (SR-B1) was found in lipid rafts and acts as a receptor for cholesterol-rich HDL. However, HDL binding to SR-B1 activates cholesterol efflux and inhibits cellular exosome absorption [80] (Fig. 2). Therefore, inhibition of internalization of pathogenic exosomes by recipient cells may be a potential therapeutic approach. Moreover, exosomes derived from melanoma could down-regulated type I interferon (IFN) receptor and IFN-inducible cholesterol 25-hydroxylase (CH25H). CH25H produces 25-hydroxycholesterol, which conversely inhibits exosome internalization [81]. As mentioned above, lipid metabolism exert a major role in the biological process of exosomes from biosynthesis to interaction with recipient cells. There is lack of systematic understanding of the biological processes of exosomes involved in lipid metabolism. However, lipid-modified exosomes promise to become new therapeutic targets.

## Disorder of lipid metabolism and diseases associated with exosomes

### Atherosclerosis

Atherosclerosis, the pathological basis of most cardiovascular diseases, is closely associated with abnormal cholesterol accumulation in the arterial intima. The formation of foam cells in the arterial intima is a major hallmark in early-stage atherosclerotic lesions, which is attributed to excessive cholesterol esterification as well as impaired cholesterol release [82, 83]. In atherosclerotic lesions, macrophages and VSMCs have been considered as the main source of foam cells [84–86]. The down-regulation of ABCA1 and ABCG1 or the up-regulation of CD36 by exosomes prevents cholesterol from being exported to extracellular acceptors apoA-I or HDL [87–89]. Circulating miRNAs in exosomes, such as miR-30e and miR-92a, can inhibit the expression of lipid transporters via binding to mRNAs 3’UTR directly [13, 62]. In addition, exosomes isolated from the supernatants of activated human CD4(+) T cells are capable of producing the pro-inflammatory cytokine TNF-α, inducing cholesterol accumulation in macrophages [90].

**Fig. 3:**
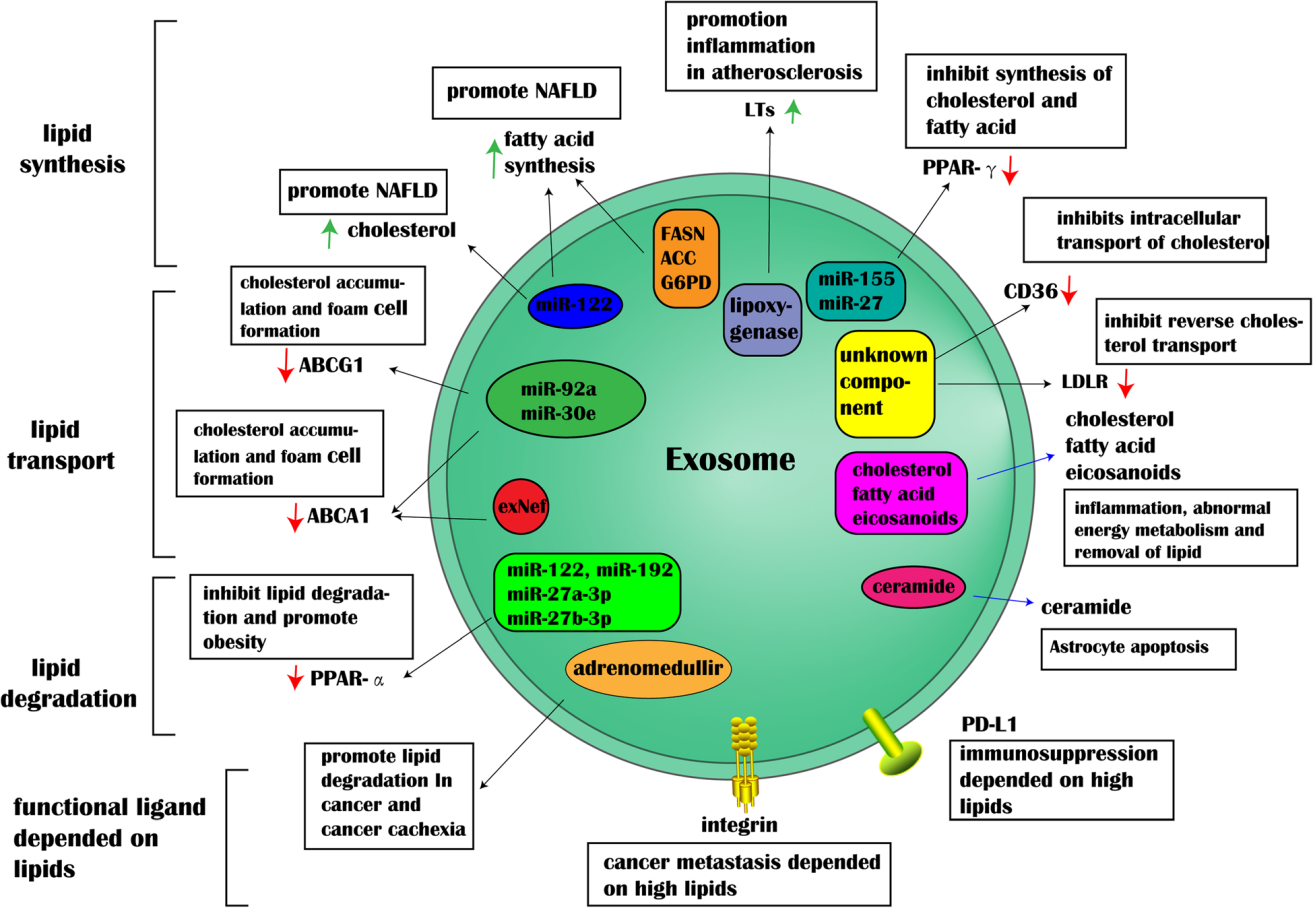
Regulation of exosomes on lipid metabolism. Exosomes regulate lipid metabolism, including lipid synthesis, transport, degradation, which involved in the development of lipid disorder diseases. In addition, functional ligands on exosomes membrane, such as PD-L1 and integrin, exert multiple effects depending on the level of lipids

It has been recognized that atherosclerosis is a chronic inflammatory disease. Some exosomes can also induce the biosynthesis of leukotrienes (LTs), which are potent pro-inflammatory lipid mediators [91–93] (Fig. 3). Esser et al. found that exosomes from macrophages and dendritic cells (DCs) contain functional enzymes for LTs biosynthesis [92]. It is known that LTA4 and LTB4 are the main product of macrophages, while DCs primarily formed LTC4. Among them, LTB4 is derived from the 5-lipoxygenase pathway of arachidonic acid metabolism and exerts a potent pro-inflammatory effect by activating G-protein coupled receptors. As a chemoattractant, LTB4 can stimulate the accumulation of monocytes on the arterial wall and assist in the differentiation of monocytes into macrophages. Exosomes derived from neutrophil also contain LTB4 and LTB4-induced enzymes, which may be involved in the recruitment process of neutrophils at the inflammation [94]. And neutrophils release chemotaxis to endothelial cells during atherosclerosis. This process may aggravate endothelial dysfunction and cause monocytes to accumulate in the vulnerable vessel walls [95].

### Cancer

Cancer is the second-leading cause of death globally, and cancers have been increasingly considered to be dysfunctions in hypoxic microenvironment [96]. It has been found that exosomes are more likely to be produced and secreted in the hypoxic cancer microenvironment, because excessive hypoxia-inducible factor-1α (HIF-1α) regulates the activation of small GTPase Rab27a (a major regulator of exosomal synthesis) [69, 97]. HIF-1α stable long non-coding RNA (HISLA) in exosomes derived from cancer-associated macrophages stabilizes HIF-1α via proline hydroxylase domain 2 (PHD2) and HIF-1α [98]. It seems that high lipid content is more conducive for normal cells to absorb cancer-derived exosomes [99, 100], and induce normal cells to transform into cancer cells [81, 101]. At present, disorder of lipid metabolism mediated by exosomes is increasingly recognized as a characteristic of cancer cells and may be a factor in the malignant cancer progression and metastatic behavior [102].

Exosomes in mediating lipid metabolism during cancer progression are getting more and more attention. Exosomes with bioactive lipids, such as PGE2α, PGE1, and PGE2, can be released from macrophages into cancer microenvironment [103, 104]. Although some cancer cells are damaged in an inflammatory state, a small fraction of cancer cells escape from a pro-inflammatory state smartly. Some chronic and uncontrolled proliferation of cancer cells are also maintained through evading immune surveillance. However, CD8 (+) T cells are the major players in removing cancer cells. Ricklefs et al. found that the exosomes derived from melanoma could inhibit the killing capacity of CD8 (+) T cells. Programmed cell death-Ligand 1(PD-L1), identified on the membrane of exosomes derived from melanoma, binds to programmed cell death protein 1(PD-1) on the surface of CD8 (+) T cells, which will cause temporary blockade of CD8 (+) T cells immunity, rather than permanent suppression [85, 86]. Thus, high cholesterol is more favorable to the binding of exosomes to CD8 (+) T cells, probably because cholesterol is enriched on the cell membrane and the fluidity of the cell membrane is also improved [105]. Meanwhile, the lipids degradation in adipose tissue can provide sufficient energy for chronic and uncontrolled cell proliferation in cancers and cancer-associated cachexia. Exosomes from Lewis lung carcinoma cells, lung carcinoma cells, and prostate cancer cell contributes to lipid degradation of adipocytes [64–66]. Furthermore, exosome-modified cancer invasion and metastasis depend on high cholesterol. Wu et al. has identified CD147 in exosomes derived from hepatocyte, which is a leading gene involved in the hepatocarcinogenesis and metastasis [99]. Recently, proteomic analysis of exosomes revealed that cancer cell-derived exosomes with different organ propensity had different integrin expression profiles, with α6β4 and α6β1 associated with lung metastasis and αvβ5 associated with liver metastasis [106]. However, Akhter et al. indicated that the cells originated from primary cancer have higher drug resistance than epithelial cell adhesion molecule (EpCAM) ^+^ cancer cells due to the over-expression of ABCA1 [107] (Fig. 3).

Mechanically, disorder of lipid metabolism also facilitates cancer invasion and metastasis by up-regulating oncogenes, such as c-Myb, c-Myc, c-Jun, cyclin-E, Notch and mTOR [100]. Roberg-Larsen et al. also confirmed that compared to exosomes derived from an estrogen receptor (ER-) breast cancer cell line (MDA-MB-231), the levels of 27-OHC in exosomes from an ER^+^ breast cancer cell line (MCF-7) has increased, which provides complementary information with diagnostic value [108]. In addition, melittin treatment can significantly increase the exosomal long-chain non-coding RNA NONHSAT105177, down-regulate of the cholesterol metabolism genes, including clusterin (CLU), and inhibit pancreatic ductal carcinoma [109]. It seems that regulation of exosome mediated-abnormal lipid metabolism may inhibit caner progression, invasion, as well as metastasis.

### Non-alcoholic fatty liver disease

Abnormal lipid metabolism is the cause of nonalcoholic fatty liver disease (NAFLD), which is the most common chronic liver disorder worldwide. The functions of exosomes have gradually become an important mechanism for the regulation of liver and disorder of NAFLD. Circulating miR-122 accounts for more than 70% of the liver miRNA pools, and contributes to lipid homeostasis in the liver [110, 111], such as fatty acid and cholesterol [54, 112]. However, the high level of miR-122 in exosomes is closely associated with NAFLD [113], because the sufficient miR-122 in hepatocytes leads to a significant increase of cholesterol and fatty acid (Fig. 3). Conversely, down-regulation of miR-122 expression triggers up-regulation of HIF-1α, vimentin, and MAP 3 K3, which can provide energy for NAFLD-induced liver fibrosis [114]. Therefore, it is necessary to further explore that miR-122-encapsulated exosomes, which are considered as a potent biological marker for hepatic carcinoma [115, 116]. Exosomes isolated from melatonin-treated adipocytes can significantly alleviate hepatic steatosis caused by high-fat diet and resistin-mediated endoplasmic reticulum stress. Melatonin reduces the levels of exogenous resistin by Bmal1 transcriptional repression and m6A RNA demethylation in adipocytes. Therefore, melatonin reduces the amount of exogenous resistin from adipocytes to hepatocytes.

### Obesity

According to WCRF statistics, obesity causes 35% of pancreatic cancers, 28% of gallbladder cancers, and 35% of esophageal cancers are attributable to obesity [117]. Meanwhile, obesity is accompanied by an increase in the amount of adipose tissue mass, and is also a major driving force for the insulin resistance and the pathogenesis of type 2 diabetes (T2D) and even metabolic syndrome. The crosstalk between adipose tissue and other tissues regulates systemic lipid metabolism through the secretion of peptide hormones, inflammatory mediators, signaling lipids, and miRNAs packaged in exosomes. Besides, obesity is closely related to the metabolic state of adipose tissue and adipose tissue-associated cells, such as macrophages [118]. Macrophages secret exosomal miR-155 and move them to adipocytes, in which miR-155 inhibits obesity by downregulating its target gene PPAR-γ [52]. In addition, circulating miRNAs in exosomes, such as miR-122, miR-192, miR-27a-3p and miR-27b-3p can also inhibit the expression of PPAR-α in white adipose tissue (Fig. 3). Mechanistically, exosomal miR-124a derived from mesenchymal stem cell can silence forkhead box A2 in macrophages, leading to disorder disturbance disorder of intracellular lipid accumulation [119]. Studies from Perez-Diaz et al. further indicated that the plasma exosomal transcription release factor (PTRF) increases the occurrence of hypertrophy and aging of 3 T3-L1 adipocyte hypertrophy and senescence. Circulating polymerase I and PTRF, as adipokines may partially contribute to the deleterious effects of visceral fat accumulation [120]. Notably, adipocyte-derived exosomes are essential for liver physiological activity [121].

### Alzheimer’s disease

Alzheimer’s disease is a kind of neurodegenerative disorder caused by the extracellular deposition of amyloid plaques formed by Aβ peptide or the intracellular deposition of tangles derived from hyperphosphorylated tau protein [122]. Several studies showed that exosomes are involved in the accumulation of Aβ through regulating lipid metabolism. However, there are two controversial views about the effects of exosomes on the accumulation of Aβ. For the brain itself, the accumulation of Aβ is eliminated by a new approach of attaching Aβ to the surface of astrocyte-derived exosome [123]. Another point of view suggests that astrocytes-derived exosomes carry ceramide and prostate-apoptotic response-4, and their accumulation results in neuronal apoptosis [1, 124, 125] (Fig. 3). In addition, overexpression of neutral sphingomyelinase is also detected in exosomes, and they are able to catalyze the production of ceramide. Lysophospholipids involved in neuroinflammation also increase with the accumulation of Aβ [126].

## Conclusion

In summary, the review covered the exosome-mediated lipid metabolism (synthesis, transportation and degradation) and its associated diseases (atherosclerosis, cancer, NAFLD, obesity, and Alzheimer’s disease). Another aspect that the present review focuses on is the involvement of lipids in the biological processes of exosomes including biosynthesis, secretion and interaction with recipient cells. Since exosomes have been isolated for more than 30 years, and the research of them provides a new perspective for better understanding of the occurrence and development of atherosclerosis, cancer, NAFLD, obesity, Alzheimer’s disease by regulating lipid metabolism. Conversely, lipid metabolism also affected the biosynthesis and secretion of exosomes as well as the physiological interaction with recipient cells. Exosomes with parental cell metabolic information and genetic information transmit signals molecules to target cells, altering lipid metabolism and promote the further progression of diseases. In addition to proteomics, exosome has been subjected to lipidomic analysis. The results indicate that exosomes derived from different pathological conditions show differences in lipids, which provides a reliable basis for the diagnosis and treatment of the diseases. Thus, exosomes could be suggested as diagnosis, prognosis and therapeutic biomarkers of diseases. A major challenge needs to be addressed is that exosomes extracted from plasma have not been fully determined by which cell secreted needs to be addressed. Although LC-MS, open tubular liquid chromatography (OTLC), liquid chromatograph-mass spectrometer/ mass spectrometer (LC-MS/MS) and multispectral optical tweezers (MS-OTs) are important approaches for the proteins detection in plasma exosomes [127, 128], more precise instruments and methods should be developed to detect lipid in exosomes. Therefore, the clinical application of exosomes needs further exploration.

## Data Availability

Not applicable.

## Abbreviations

ABCA1: ATP-binding cassette transporter A1
ABCG1: ATP-binding cassette transporter G1
LDLR: low density lipoprotein receptor
PPARs: peroxisome proliferators-activated receptors
FASN: fatty acid synthetase
NAFLD: non-alcoholic fatty liver disease
HSP: heat shock protein 70
MVBs: multi-vesicular bodies
PDGFR: platelet-derived growth factor receptors
EGFR: endothelial growth factor receptors
ACC: acetyl-CoA carboxylase
G6PD: glucose-6-Phosphophosphate dehydrogenase
GPI: glycosylphosphatidylinositol
RCT: Cholesterol reverse transport
VSMCs: vascular smooth muscle cells
exNef: HIV-1 protein Nef
HFD: high-fat diet
hAD-MSCs: adipose tissue-derived mesenchymal stem cells
ILVs: intraluminal vesicles
ESCRT: endosomal sorting complex required for transport
PA: phosphatidylcholine
PLD2: phospholipase D2
DGK: diacylglycerol kinase
SR-B1: scavenger receptor type B-I
IFN: interferon
CH25H: 25-hydroxylase
LTs: leukotrienes
HIF-1α: hypoxia-inducible factor-1α
PHD2: proline hydroxylase domain 2
PD-L1: Programmed cell death-Ligand 1
PD-1: programmed cell death protein 1
EpCAM: epithelial cell adhesion molecule
ER: estrogen receptor
PTRF: plasma exosomal transcription release factor
OTLC: open tubular liquid chromatography
LC-MS/MS: liquid chromatograph-mass spectrometer/ mass spectrometer
MS-Ots: multispectral optical tweezers
